# On the Use of Biobased Waxes to Tune Thermal and Mechanical Properties of Polyhydroxyalkanoates–Bran Biocomposites

**DOI:** 10.3390/polym12112615

**Published:** 2020-11-06

**Authors:** Vito Gigante, Patrizia Cinelli, Maria Cristina Righetti, Marco Sandroni, Giovanni Polacco, Maurizia Seggiani, Andrea Lazzeri

**Affiliations:** 1 Inter University Consortium of Material Science and Technology, c/o Unit Department of Civil and Industrial Engineering, University of Pisa, Largo Lucio Lazzarino 1, 56122 Pisa, Italy; vito.gigante@dici.unipi.it (V.G.); info_lam@katamail.com (M.S.); giovanni.polacco@unipi.it (G.P.); andrea.lazzeri@unipi.it (A.L.); 2 CNR-IPCF, National Research Council—Institute for Chemical and Physical Processes, Via Moruzzi 1, 56124 Pisa, Italy; cristina.righetti@pi.ipcf.cnr.it

**Keywords:** biobased waxes, poly(3-hydroxybutyrate-co-3-hydroxyvalerate), natural fillers, wheat bran

## Abstract

In this work, processability and mechanical performances of bio-composites based on poly(3-hydroxybutyrate-co-3-hydroxyvalerate) (PHBV) containing 5, 10, and 15 wt % of bran fibers, untreated and treated with natural carnauba and bee waxes were evaluated. Wheat bran, the main byproduct of flour milling, was used as filler to reduce the final cost of the PHBV-based composites and, in the same time, to find a potential valorization to this agro-food by-product, widely available at low cost. The results showed that the wheat bran powder did not act as reinforcement, but as filler for PHBV, due to an unfavorable aspect ratio of the particles and poor adhesion with the polymeric matrix, with consequent moderate loss in mechanical properties (tensile strength and elongation at break). The surface treatment of the wheat bran particles with waxes, and in particular with beeswax, was found to improve the mechanical performance in terms of tensile properties and impact resistance of the composites, enhancing the adhesion between the PHBV-based polymeric matrix and the bran fibers, as confirmed by predictive analytic models and dynamic mechanical analysis results.

## 1. Introduction

The progressively growing activities related to environmental sustainability leads to a continuous search for knowledge, even in the field of polymeric materials. One of the elements supporting this ’green revolution’ concerns the development of bioplastics and bio-composites, which represent an important contribution to the decarbonization of the economy, without renouncing the convenience of evolution [1].

Among the commercially available bioplastics, polymers of the family of polyhydroxyalkanoates (PHAs) are noteworthy due to their excellent biodegradability in different environments (industrial/home composting, soil, fresh water and sea water), thermoplastic properties, good mechanical properties, and biocompatibility.

PHB (poly-hydroxybutyrate) and PHBV poly(3-hydroxybutyrate-co-3-hydroxyvalerate) are the most used PHA polyesters synthesized and stored by various microorganisms as an intracellular reserve of carbon and energy, starting from sugar and oil vegetables, under unbalanced conditions [2]. Specifically, PHBV is obtained by incorporation of monomeric units of hydroxyvalerate (HV) along the PHB chain which leads to distortion in the homopolymer crystalline lattice with consequent reduction of the crystallinity degree [3]. Therefore, the introduction of HV units provides more flexibility and ductility compared to PHB, consequently widening the range of applications and making processing more viable.

PHBV shows good stiffness and crystallinity that guarantee good thermal resistance [4] but low barrier to water [5]. Moreover, these polymers are highly biodegradable, they can be assimilated and degraded by a great variety of microorganisms, thus avoiding the accumulation of plastic waste in the environment [6]. Nevertheless, PHBV has some limits such as low impact resistance and tensile brittleness [7]; and the relatively high cost [8], compared to other bioplastics such as polylactic acid (PLA), has hampered its use in basic applications such as packaging and service items.

With the intention of increasing the use of this microbial polyester, lowering the cost of the final product, PHBV is increasingly combined with natural fillers and fibers [9,10], which have been proved to be effective in promoting disintegration and biodegradation of the biocomposites when disposed for biorecycling [11,12]. For this reason, various studies have been carried out on the production of biocomposites incorporating low-value materials or food waste by-products into PHBV, such as waste lignocellulose fibers, highly-available at low-cost, from agricultural and industrial crops [9,13,14,15,16], Many types of natural fibers were investigated, such as wood dust, hemp, feather, kraft pulp, jute, flax, sisal fibers, etc. [17]. However, there is an ongoing search for new fibers or fillers for bio-composites. For example, there are large amounts of grain by-products, with low cost and wide availability—such as wheat bran, straw, corn stalk, and rice husk—which could be used for producing biodegradable composites.

Bran is the main by-product of flour milling; it is used only for 10% in bakeries and in breakfast cereals as a dietary fiber supplement and the remaining 90% could be sold as animal feed, but millers often dispose of the bran as waste, due to high transport costs, with consequent environmental risk. Wheat bran contains starches, phenolic compounds, soluble and insoluble dietary fibers and proteins [18]. The water insoluble fraction of bran consisting of cellulose (~21%), hemicellulose (~26%), and lignin (~5%) [19] can offer advantages as reinforcement material [20]. Biomass fibers derive their strength from the hydrogen bond in microfibrils and, in general, the increase in the cellulose fraction increases the strength of the fibers [21,22,23].

The use of lignocellulose fibers as a reinforcement in polyester matrices such as PHBV requires the issue of compatibility between the two phases to be addressed in order to obtain composites with good mechanical performances. In fact, the natural lignocellulose fibers exhibit a poor compatibility with polyester matrices due to a different hydrophilicity of their surfaces [24,25]. As a consequence, the mechanical properties of the composite are severely reduced. When a material is subjected to an external load, the matrix transfers a part of the load by applying a shear stress, through the interface, on the dispersed phase; therefore, a strong adhesion between the phases results in a better distribution of the applied stress and, consequently, in a high resistance and stiffness of the composite [26].

In addition, the typical hydrophilicity of the fiber, in contrast to the more hydrophobic nature of many polymeric matrices, can induce, by water absorption, the formation of cracks and consequently undermine the strength of the composite [27].

However, the poor compatibility between fiber and polymeric matrix can be improved by modifying the fiber surface by physical and/or chemical methods [28]. For example, a pre-treatment with plasma can induce compatibility between hydrophilic fibers and hydrophobic matrices, through the formation of free radicals and crosslinking of the fibers surfaces. Regarding chemical treatments, Wei et al. [29] improved the mechanical resistance of PHBV grafting it on the cellulose fibers by reactive extrusion with dicumyl peroxide; moreover, Alam et al. [30] produced composites with optimized ultrasound treated oil palm (EFB) fibers treated with alkali solution concentration, hyper branched polyester solution, exposing time and temperature and a significant increase in mechanical and interfacial properties was found. Herrera-Franco et al. [31] deposited a silane coupling agent to henequen fibers and have shown that adhesion between the natural hard fibers and matrix plays an important role on the final mechanical properties of the composite. Vilay et al. [32], in their research, even bagasse fibers have been exploited as reinforcing component for unsaturated polyester resin. The chemical treatments using sodium hydroxide (NaOH) and acrylic acid (AA) were carried out to modify the fiber properties. At different fiber loadings, AA treated fiber composites show better mechanical properties compared to those of NaOH treated fiber composites. SEM investigations show that the surface modifications improve the fiber–matrix interaction.

Righetti et al. [33] treated potato pulp powder with biobased waxes to improve the compatibility with PLA. Indeed, the waxes, by coating the fiber, reduce its hydrophilicity and, at the same time, increase its surface roughness improving its adhesion to the polymeric matrix. Good outcomes have been achieved thanks to the waxes that guaranteed improved dispersion of the fibers and greater adhesion with a polyester matrix. Those results, for the present manuscript, were taken as a starting point, given the similarity of the matrix (in fact, Arrieta et al. [34] stated that PLA and PHBV show similarity in melt processing).

Oil-based paraffin waxes, although non readily biodegradable [35], are often used as anti-caking additives, showing excellent moisture barrier properties. In addition, they can improve also the anti-blocking properties of a composite, because generally the natural fibers tend to clump together under pressure and heat [36].

The aim of the present work was to develop bio-composites based on PHBV and wheat bran (5, 10, and 15 wt %), pre-treated with non-ionic aqueous emulsion of carnauba wax and beeswax in order to improve the adhesion between PHBV matrix and natural fibers and their dispersion in the polymeric matrix. The composites were processed by twin-screw extruder and, then by injection molding. The objective was to investigate if also with the system PHBV/bran could have a similar behavior of other biocomposite systems [33] with the addition of biobased waxes.

Thermal, morphological, rheological, mechanical, and dynamical–mechanical–thermal analyses were carried out and predictive models were applied to characterize the developed bio-composites and to compare the effect of fiber treatment performed with the two different types of biobased waxes.

## 2. Materials and Methods

### 2.1. Materials and Processing

- PHBV (PHI002), by NaturePlast^®^ (Caen, France), was used as matrix for the biocomposites. It is a polyhydroxybutyrate having 5 wt % of valerate content with a density of 1.25 g/cm^3^. It is a thermoplastic polymer with high crystallinity, due to his isotactic structure. Supplier data sheet reports a glass transition temperature, *T*_g_, evaluated with differential scanning calorimetry (DSC), of 5 °C and a melting temperature, *T*_m_, of 175 °C.
- Wheat bran was received ground by Barilla (Parma, Italy) and sieved with 250 μm mesh. The starch content of the sample used varies between 10 and 18%, also in accordance with literature data [37]. Bran was dried in a ventilated oven at 110 °C for about 24 h.
- Acetyl tributyl citrate (ATBC), supplied by Tecnosintesi^®^ (Bergamo, Italy), was used as plasticizer. It is a biobased, biodegradable, colorless, odorless, and organic liquid. It has a density of 1.05 g/cm^3^ and a molecular weight of 402.5 g/mol.
- Calcium carbonate, CaCO_3_, (OMYACARB 1-AV) purchased from Omya^®^ (Avenza/Carrara, Italy) was used as inorganic filler (average particle diameter of 1.6 μm) for guaranteeing the sample removal from the mold in the injection molding.
- Aquacer T561 (non-ionic aqueous emulsion of beeswax) and Aquacer T581 (non-ionic aqueous emulsion of carnauba wax) purchased from BYK (Wesel, Germany) were used to wet bran fibers to improve their adhesion with the matrix and dispersion in the matrix.

The treatment with biobased waxes of the bran fibers, previously ground and sieved, was carried out wetting the bran with a dilute emulsion of wax in demineralized water: emulsified wax (carnauba or beeswax) ratio of 2:1 (*v*/*v*), and maintaining a wetting ratio (g of emulsified wax: g of dry fiber) of 1:20. The wetting process was conducted in knives blender operating at high speed, slowly injecting the emulsion of wax.

The water/wax ratio of 2:1 was chosen by evaluating the emulsion stability threshold as the dilution ratio increases. The wetted fiber was then reintroduced into the blender and remixed to obtain a product completely free of aggregates. No differences in volumetric fraction between treated and untreated bran with biobased waxes have been detected.

The wetted bran was kept in a ventilated oven at 110 °C for 48 h and, then, processed in a co-rotating twin-screw extruder (EBC25HT, Comac s.r.l, Cerro Maggiore, Milan, Italy) with two 25 mm co-rotating screws in a barrel system with L/D = 44 for producing granules of PHBV/bran composites at different composition. PHBV granules were fed by the main hopper, the bran fibers (untreated or treated with waxes) and calcium carbonate were fed by a side K-Tron Coperion hopper (Milan, Italy); while the ATBC plasticizer was fed into the extruder at two-thirds of the screw length by using a Verderflex peristaltic pump (Castleford, UK) equipped with a silicon tube which was previously calibrated to control the flow rate.

The temperature profile adopted for all the composites was 150/165/170/170/170/175/175/175/175/175/175 °C, with the die exit zone at 175 °C. The screws rate was kept at 300 rpm and the mass feed at 15 kg/h.

The exit strands were cooled in cold water bath and dried by a constant jet of air and, then, pelletized in a mechanical cutter. The resultant granules were dried for 8 h in a dryer at 50 °C (Piovan S.p.A., Venice, Italy) and, then, used to obtain ISO 527 1-A dog bone specimens and ISO 179 parallelepipedal specimen by an injection molding machine (Megatech H18/10) for the subsequent tensile and impact tests, respectively. The temperature profile used was the following: 165/170/175 °C. The mold was kept at 50 °C for 10 s with an injection pressure of 120 bar. In Table 1 the composition (wt %) of the developed composites is reported.

### 2.2. Testing Methods

Thermogravimetric analyses (TGA) were performed using a TA Q-500 (TA Instruments, Waters LLC, New Castle, DE, USA). About 15 mg of the sample to be analyzed was put into a platinum pan and heated from room temperature to 700 °C at 10 °C/min under nitrogen atmosphere.

Differential scanning calorimetry (DSC) measurements were performed with a TA Q200 equipped with a refrigerating system. The instrument was calibrated in temperature and enthalpy according to the procedure for standard DSC. Dry nitrogen was used as purge gas at a rate of 50 mL/min. The as prepared samples were analyzed from −50 to 200 °C at the heating rate of 10 °C/min.

The effect of bran loading on the fluidity of the biocomposites was evaluated by the melt flow rate (MFR) and melt volume rate (MVR) measurements, carried out according to UNI EN ISO 1133 by a CEAST Melt Flow Tester MF20 (Instron, Canton, MA, USA). MFR and MVR represent the content of melt polymer, in mass (g) and volume (cm^3^), flowing per 10 min through a capillary of specific diameter and length under a pressure applied at a specified temperature. A 5 g measure of sample pellets were heated at 190 °C in the barrel and extruded through the normalized die (2.095 mm) under a constant load of 2.16 kg. Melt volume rate (MVR) value has been evaluated as a function of time; more specifically the transition of the polymeric melt within a known volume (the one of the nozzles of the melt flow tester) has been recorded every 5 s to understand if the fluidity of the melt is affected by the increase in residence time at working temperature. The curves shown in the figure are average curves of at least five tests carried out for each material.

Tensile tests were carried out on ISO 527 1-A dog bone specimens at room temperature and at a crosshead speed of 10 mm/min by an Instron 5500R universal testing machine (Canton, MA, USA) equipped with a 1 KN load cell and interfaced with a computer running MERLIN software (version 4.42S/N-014733H). At least five specimens for each formulation were tested and the average values were reported.

Impact tests were performed on ISO 179 V-notched specimens (V-notch 2 mm at 45°) at room temperature using a 15 J Charpy pendulum of an Instron CEAST 9050 (Canton, MA, USA). The standard method ISO179:2000 was followed. For each formulation, at least five specimens were tested and the average values were reported.

In all the described experimental tests the variation with respect to the mean value and the error bars of the graphs in this work are obtained using the following standard deviation formula

(1) $$\sqrt{\frac{\Sigma \left(x-\bar{x}\right)}{n-1}}$$

where x is the sample mean value and *n* is the sample sizing.

Dynamic mechanical thermal analysis (DMTA) to evaluate the adhesion factor was performed by a Gabo Eplexor^®^ 100N (Gabo Qualimeter GmbH, Ahlden, Germany) in tensile configuration on specimens having dimensions of 40 × 10 × 1 mm^3^ (obtained by cutting in the central part (useful section) of the dog bone injection molded specimens) in the temperature range from −30 to 60 °C with heat rate of 2 °C/min and at frequency of 1 Hz.

Finally, to investigate the microstructure, the dimensional distribution of grinded bran, the distribution of bran fibers in the PHBV matrix and the matrix/filler adhesion, the bran powder and cryo-fractured composites were analyzed by scanning electron microscope (SEM) FEI Quanta 450 FEG (ThermoFisher, Waltham, MA, USA) equipped with a large field detector for low kV imaging simultaneous secondary electron (SE). The samples were previously gold sputtered by using a sputter coater Edward S150B.

## 3. Results and Discussion

### 3.1. Thermogravimetric Analysis

**Figure 1:**
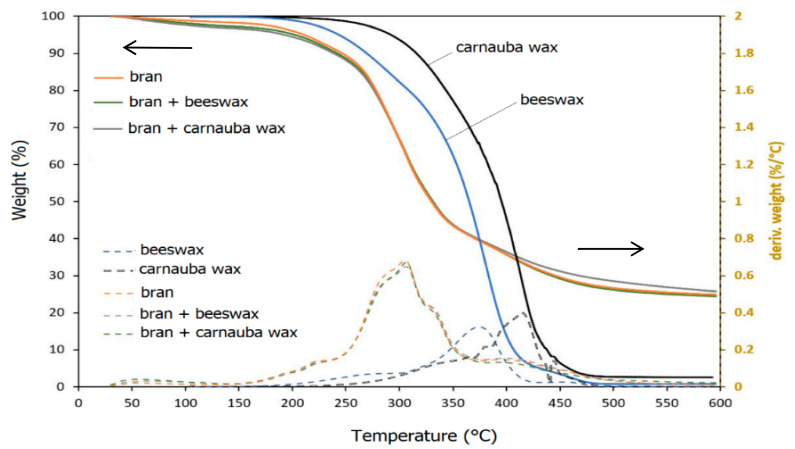
TG and DTG of the untreated and treated bran fiber beeswax and carnauba waxes in nitrogen.

Figure 1 shows the thermogravimetric (TG) and derivative thermogravimetric (DTG) curves of the bran fibers, untreated and treated with beeswax and carnauba emulsion, and beeswax and carnauba wax obtained by drying at 105 °C of relative emulsions.

The bran fibers showed a first weight loss (about 5%) at temperature lower than 105 °C, corresponding to water loss and then, several degradation steps were observed. The first thermal decomposition began at a temperature of about 190 °C with a peak temperature of 300 °C; then, the decomposition continued up to 500 °C showing a second small peak at 400 °C, remaining about 25% of residue (char and ash). Wheat bran contains significant amounts of cellulose, hemicellulose, lignin, and other compounds (e.g., proteins, starch), according to literature. The decomposition of hemicellulose and lignin occurs in the lower temperature range, instead the decomposition of the cellulose, resulting in the formation of volatiles and char, occurs at higher temperatures.

As shown, there are multiple weight losses also in the DTG curves of beeswax and carnauba wax which agrees with their multi-component composition (fatty acids, primary and secondary alcohols, ketones, aldehydes, fatty acid esters, etc.). Both waxes started to lose weight around 200 °C and resulted almost completely decomposed when the temperature reached 480 °C, remaining a residue below 2%. The beeswax showed higher degradation rate compared to carnauba wax in the range 200–390 °C.

Due the very low concentration of the waxes in the treated bran (below 1%), no difference was observed between the TG and DTG curves of the treated bran and those of untreated ones.

Consequently, the thermal stability of the bran fillers (treated and untreated) and of the used waxes resulted to be suitable for the PHBV processing temperature range (160–175 °C), noticing also that the amount of residual moisture of the dried fiber covered with waxes is between 2 and 2.5%.

### 3.2. Morphology Analysis

**Figure 2:**
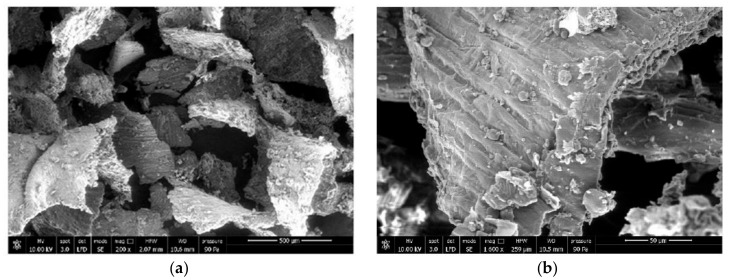
SEM images at (**a**) 200× and (**b**) 1600× of bran smaller fraction.

Figure 2 show SEM images of the bran by-product used. Most of the bran consists of flakes with a low aspect ratio and dimensions mainly in the range of 250–500 μm and even smaller size fractions are observed. Indeed, the process of separation of bran from the seed core produce different fractions of different size, composition, and content of lignocellulosic material [37].

The surface morphology of wheat bran displayed, microstructurally, the presence of protein, starch, fat, and globular particles. The presence of a smooth waxy surface over bran leaf, called cuticle, identified as aliphatic wax [38], was evident.

**Figure 3:**
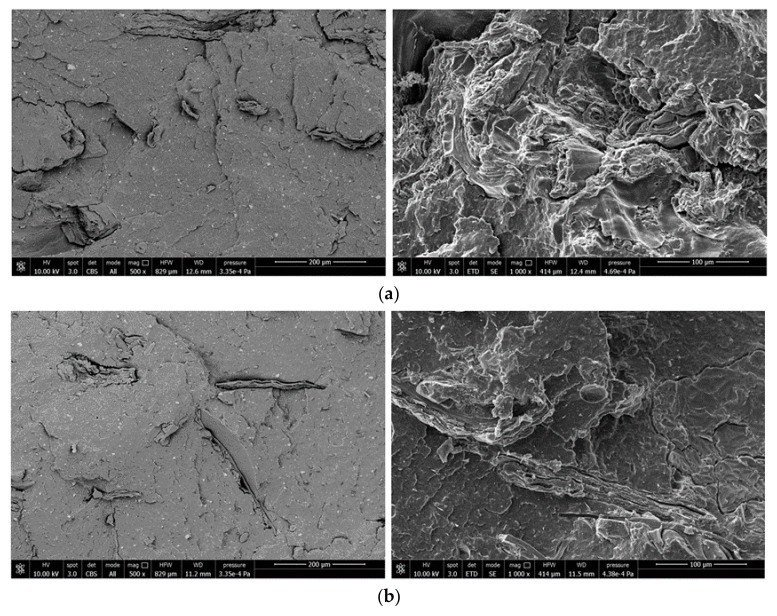

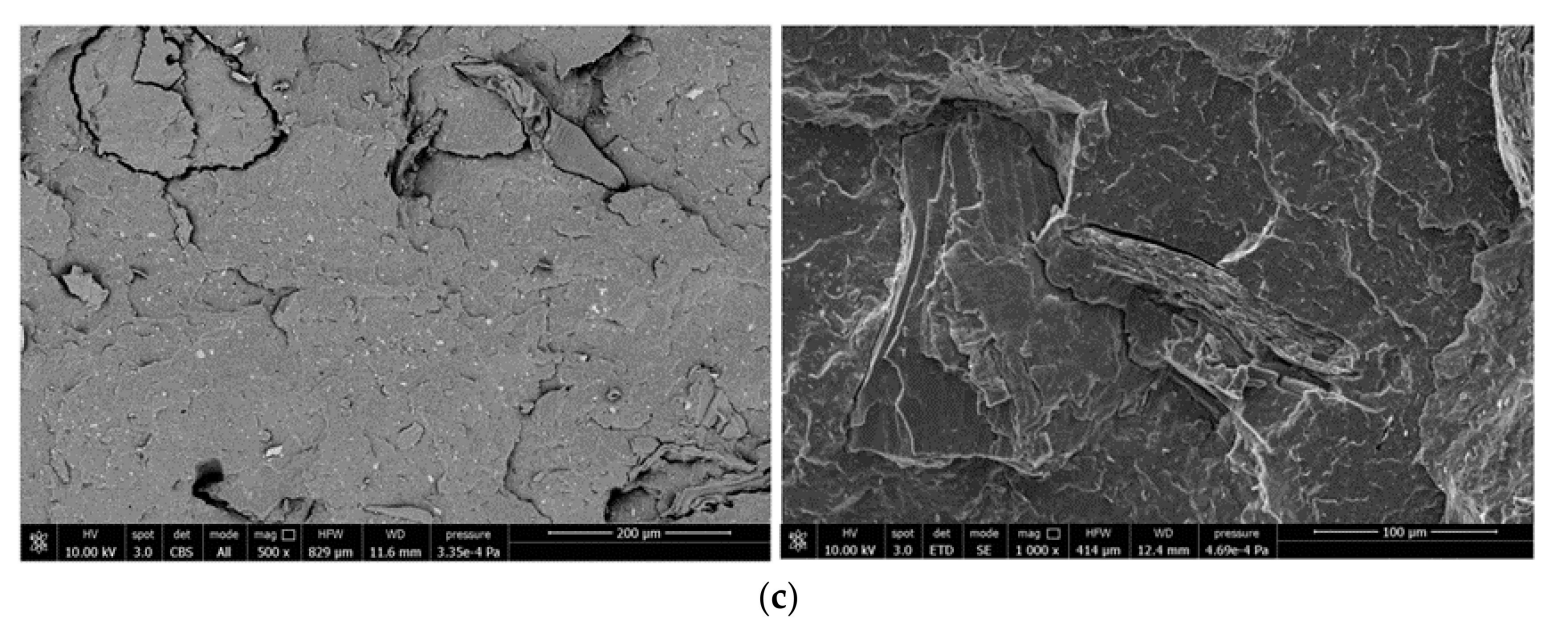
SEM-images of composites containing (**a**) 10% untreated bran; (**b**) 10% bran filler treated with beeswax (T561) and (**c**) 10% bran filler treated with carnauba wax (T581).

Figure 3a–c show, as example, back scattering SEM images (500× and 1000×) of the cryo-fractured surfaces of the composites containing 10 wt % of untreated bran and bran treated with the waxes.

A homogeneous distribution of bran flakes, whose length is in the order of 300/400 microns, is observed with absence of aggregates. In terms of filler/matrix adhesion the surface in Figure 3a appears smoother and this could be a first symptom of different fracture mechanism and therefore different adhesion between treated and untreated filler.

### 3.3. Melt Flow Analysis

**Figure 4:**
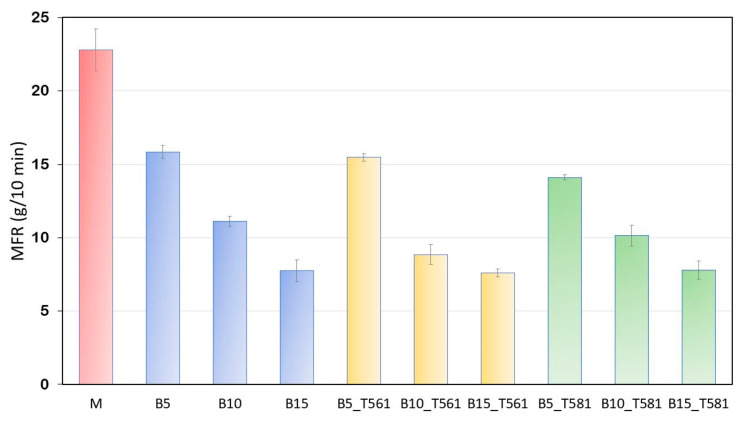
MFR values for the developed composites containing untreated and treated bran powder.

From a processing point of view, it is of great interest to know the fluidity of the molten material and the effect of the bran filler (treated with waxes and not) on the MFR and MVR to predict the behavior of the composites during extrusion and molding. Figure 4 shows how the matrix fluidity was much higher than that of all composites, showing that the bran gave strength to the melt.

**Figure 5:**
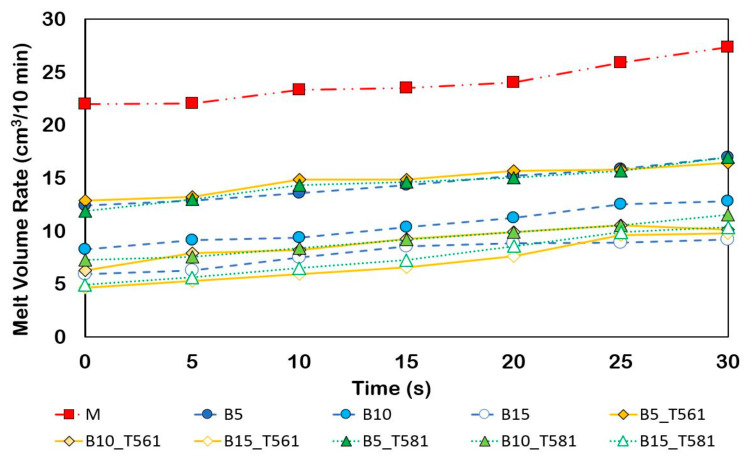
MVR values for the developed composites containing untreated and treated bran powder over time.

As the content of the filler, both treated with wax and untreated, increased, the flow resistance increased. Slight downward deviation of the values of composites processed with T561 (beeswax), thus showing a more viscous flow behavior. Interesting is the graph of the MVR (Figure 5) measured in a time interval of 30 s, thus simulating a typical residence time within extrusion process [39]. The time that the polymer stays inside an extruder as a physically and chemically active hot melt is the “effective residence time” [40].

As shown in Figure 5, a slight increase of the fluidity index with time is observed, probably due to the triggering of degradative processes. However, the increase is very limited showing that all the composites developed were stable in the so-called ’efficient residence time’ of extrusion processes.

### 3.4. Mechanical Properties

**Figure 6:**
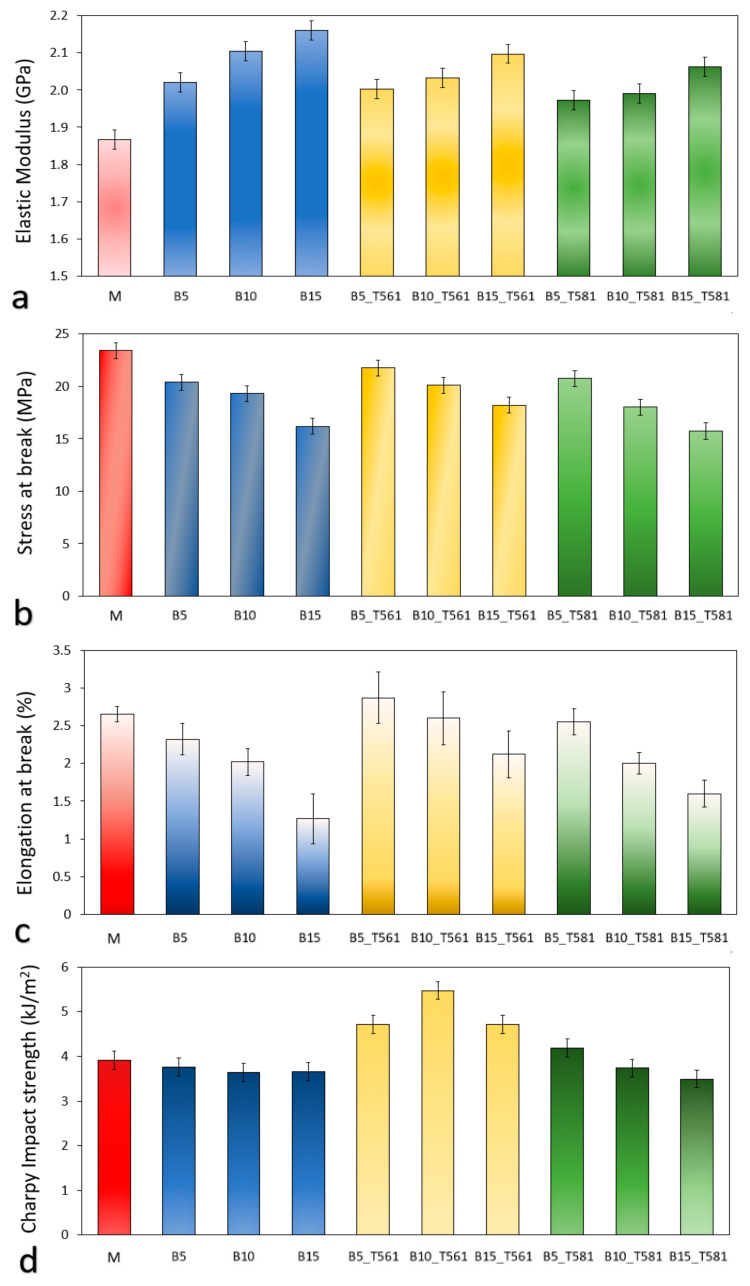
Mechanical properties of PHBV/bran composites: (**a**) elastic modulus, (**b**) stress at break, (**c**) elongation at break, and (**d**) Charpy impact strength.

Figure 6a–d reports the mechanical properties of the produced composites derived from quasi-static uniaxial tensile test and Charpy impact test.

As shown in Figure 6a, the stiffness of the composites increased as the bran fiber load increased, as reported in several works where natural fibers have been added to bio-polyester matrices [41,42,43]. The increase in stiffness is almost linear for the composites with untreated bran; while, for the composites with the filler treated with waxes, going to flatten, indicating that waxes act as a slider due to their oily consistency.

The treatment with carnauba wax and beeswax caused a lowering of stiffness accompanied by an increase in elongation at break (Figure 6c). Consequently, the composites containing bran treated with the waxes turned out less brittle than the ones with untreated bran.

Together with the increase in stiffness, a reduction of the break strength with bran content was observed (Figure 6b), suggesting a poor interfacial interaction and consequent inefficient load transfer between polymeric matrix and filler [28,44]. Poor interaction due to low compatibility is typical when natural fibers are incorporated into polymeric matrices [45,46,47], obtaining composites with lower tensile strength compared to the neat matrix but with higher elastic modulus.

**Figure 7:**
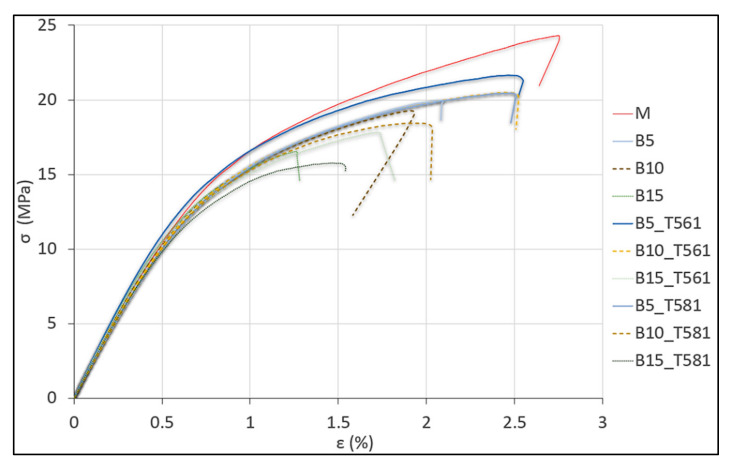
Stress–strain curves of PHBV-based biocomposites.

These assessments are clearer from the visual analysis of the experimental stress–strain curves in Figure 7.

Figure 6d shows that the composites containing untreated bran, independently of the loading, presented values of Charpy impact strength around 4 kJ/m^2^, similar to that of the unfilled-matrix, but with beeswax (T561) treatment, the resistance to impact increased 25% respect to the unfilled-matrix for the composites B5_T561 and B15_T561 and of 40% for B10_T561. Thus, the biocomposite with 10% bran, treated with beeswax, can be considered the best performing given its high impact resistance (5.5 ± 0.3 kJ/m^2^) associated to a valuable stiffness (2.0 ± 0.2 GPa) and break resistance (21.2 ± 1.3 MPa).

#### Predictive Models for the Mechanical Properties

To better interpret the data of the mechanical tests and investigate the adhesion between bran filler and the polymeric matrix, analytical models based on static and dynamical mechanical tests were applied.

Factors such as particle size, particle/matrix interfacial strength and particle loading strongly affect the stress transfer between matrix and filler [48]. The slight decrease in the maximum strength with the filler content, evidenced in Figure 6b, can be due to a negligible stress field around the bran particles. Break stress of composites containing natural fibers is usually constrained among two limits called upper and lower bound [49]. In particular, if there is no adhesion between filler and the polymeric matrix, the load is supported only by the matrix and the Equation (2) was proposed to predict the composite strength [50]

(2) $$\sigma_{c}=\sigma_{m}\left(1-1.21\cdot V_{p}^{2/3}\right)$$

where $\sigma_{c}$ and $\sigma_{m}$ are the strength of the composite and the matrix, respectively, and *V_p_* is the volume fraction of filler, evaluated as the volume fraction of bran filler compared to the base matrix consisting of PHBV, calcium carbonate and plasticizer. The calculated $\sigma_{c}$ values represent the lower bound reported in Figure 7. Contrarily, when a good adhesion between matrix and filler is guaranteed, the composite strength is given by Equation (3) [51]

(3) $$\sigma_{c}=\sigma_{m}\left(1-V_{p}\right)$$

**Figure 8:**
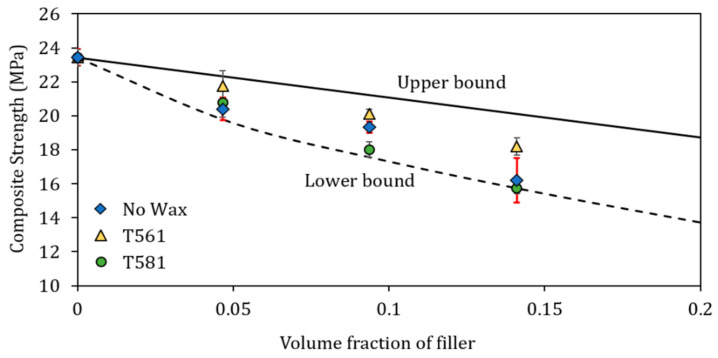
Comparison between the experimental composite strength and the values predicted according to the upper and lower bound equations.

The so-calculated $\sigma_{c}$ values represent the upper bound reported in Figure 8. As shown, the experimental data are positioned between the two limits. In particular, it can be seen that the untreated bran series (no wax) and the series with bran treated with carnauba wax (T581) are mostly characterized by lower break stress and their experimental data are positioned near the lower bound indicating lesser matrix/filler adhesion compared to the series with bran treated with beeswax (T561).

In order to better investigate the matrix/filler adhesion, the Pukánszky’s model [52] was used where the reinforcing effect of filler is expressed quantitatively by considering the effect of the decrease in effective load-bearing cross-section of the polymer (Equation (4))

(4) $$\ln\sigma_{c,red}=\ln\frac{\sigma_{c}\left(1+2.5\;V_{p}\right)}{1-V_{p}}=\ln\sigma_{m}+BV_{p}$$

$\sigma_{c,red}$ is the reduced tensile strength, i.e., the tensile strength normalized to the cross-section perpendicular to the load direction, $\sigma_{c}$ and $\sigma_{m}$ are the break stress of the composite and the matrix, respectively, *V_p_* is the filler volume fraction, and *B* is a parameter connected to the matrix/filler interaction. From the slope of the logarithm of $\sigma_{c,red}$ against *V_p_*, the value of the B parameter can be evaluated. *B* has no direct physical meaning, but it is connected with the interfacial properties of the system. In a simplified way, the higher B is, the better the adhesion.

**Figure 9:**
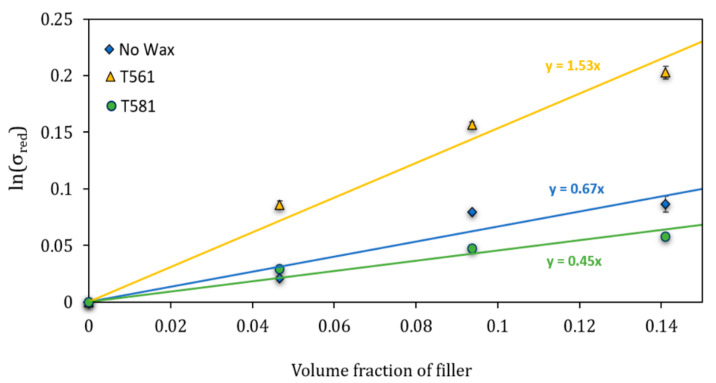
Reduced tensile strength as a function of the filler volume fraction for the determination of Pukanszky’s B parameter.

The results of the Pukanszky’s model are reported in Figure 9. As shown, the treatment of bran filler with beeswax (T561) ensured much better adhesion than that of the other two series of composites because the slope of the trend line (which represents the Pukanszky’s B parameter) is significantly higher. These results confirmed the better mechanical performances of the composites containing bran treated with beeswax.

### 3.5. DSC Analysis

**Figure 10:**
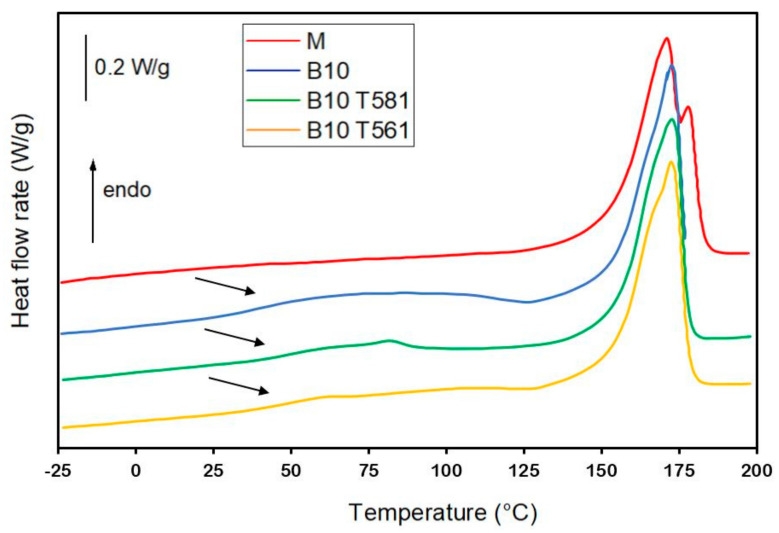
Heat flow rate curves of the as prepared matrix and composites with 10% of untreated and treated bran fibers. The curves were obtained upon heating at 10 K/min after previous cooling to −50 °C.

The thermal properties of the as prepared polymeric matrix and the composites with 10 wt % of bran, treated and untreated with waxes, were investigated by DSC to determine if the filler influences the crystallinity of the material. The heat flow rate curves, obtained at 10 K/min, are shown in Figure 10. The curves of Figure 10 have been slightly enlarged and arrows have been added to mark the endothermic event most likely connected to the enthalpy recovery subsequent to the structural relaxation of the RAF.

The glass transition of PHBV, which occurs in proximity of 5 °C, in agreement with literature data [53], is scarcely visible due to the high crystallinity of the samples: The endothermic event that is observed especially in the heat flow curves of the composites, at temperatures higher than 25 °C, has to be connected to a process of enthalpy recovery subsequent to the structural relaxation of the rigid amorphous fraction, which occurs at room temperature [54]. This event is much more marked in the composites probably due to a higher amount of rigid amorphous fraction located at the polymer/filler interface [55]. The melting process of beeswax (T561) and the carnauba wax (T581) are barely visible at approximately 60 °C and 80 °C, respectively [16]. The melting of PHBV extends from approximately 125 °C to 180 °C, and shifts to slightly lower temperatures in the composites, due to a lower perfection of the PHBV crystals that grow in the presence of the filler. A multiple melting behavior is exhibited by all the samples, which indicates the reorganization and recrystallization upon heating [56].

Table 2 lists the enthalpy of fusion (Δ*h_m_*) values measured from the heat flow rate curves of the as prepared samples (Figure 10) normalized to the PHBV content, and the crystalline weight fraction (*w_C_*) calculated from the Δ*h_m_* values divided by the enthalpy of fusion of 100% crystalline PHBV, assumed equal to that of the homopolymer PHB (Δ*h_m_* = 143 J/g) [56].

The *w_C_* value of the matrix and the composites containing bran, treated and untreated with waxes, are close and within the experimental error. This result attests that the bran, also after surface treatment with waxes, do not act as nucleating agents for the crystallization of PHBV.

### 3.6. DMTA Analysis

DMTA characterization was carried out to investigate the effect of bran loading and the superficial treatment with waxes on the viscoelastic properties of the composites. The DMTA measurements were carried out in the range from −25 to 60 °C to evaluate the glass transition temperature, *T*_g_, and the drop of storage modulus, E’, with increasing temperature.

**Figure 11:**
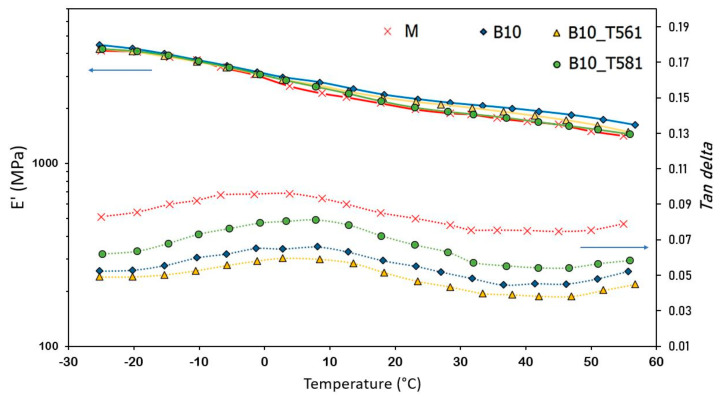
Storage modulus and tan δ trend vs. temperature for composites with 10% of untreated and treated bran.

This analysis was limited to the composites with 10 wt % of bran, treated and untreated with waxes, which exhibit the same crystallinity degree and better mechanical performances. Figure 11 shows the evolution of the storage modulus and Tan δ (ratio between elastic and viscous response to a sinusoidal stress to which the specimen is subjected) of the composites as a function of temperature, using a heating rate of 2 °C/min and an oscillation frequency of 1 Hz.

*T*_g_ values, calculated from the peak maximum of the Tan δ [57], are reported in Table 3. As shown, the presence of 10% bran increased slightly the *T*_g_ of the polymeric matrix from about 4 to 8 °C. In Table 2, the storage modulus at 25 °C is also reported, which resulted consistent with the values obtained by the tensile test (Figure 6a).

In addition, the effect of an interphase region on the dynamic properties can be quantified noticing that strong interactions between filler and matrix at the interface tend to reduce the macromolecular mobility near the filler surface compared to that in the bulk matrix [58]. This effect reduces tan *δ*, and an analytical model was developed to evaluate the so-called adhesion factor, A, in terms of the relative damping of the composite and the polymer matrix and the volume fraction of the filler as follows (Equation (5)) [59]:

(5) $$A=\left(\frac{1}{1-V_{p}}\right)\left(\frac{\tan\delta_{c}}{\tan\delta_{m}}\right)-1$$

where the subscripts *c* and *m* denote composite and matrix, respectively. High level of interface adhesion between matrix and filler particles reduces the molecular mobility surrounding the filler and this reduces $\tan\delta_{c}$ values and consequently low values of A are obtained [60]. The A values calculated for the base and composites with 10 wt % bran are reported in Table 3. As shown, the A values are in line with the results achieved by Pukanszky’s model, which indicates the highest matrix/filler adhesion for the composites containing bran filler treated with beeswax. Negative values of adhesion factor are allowed because neglecting the filler has a slight influence on the macromolecular mobility at the filler surroundings [61].

## 4. Conclusions

In a context of circular economy, wheat bran, a by-product of the agri-food industry, was used as filler in biodegradable composites based on PHBV. Surface treatments of bran filler with biobased waxes (carnauba wax and beeswax) were investigated to improve the matrix/filler adhesion and, consequently, the mechanical performances of the produced composites.

The results were satisfying in terms of processing and mechanical properties up to 15% by weight of bran content. In particular, the bio-composites with wheat bran treated with beeswax showed the best mechanical performance in terms of impact resistance (5.5 ± 0.3 kJ/m^2^) associated to a valuable stiffness and break resistance. This is attributable to the best matrix/filler adhesion observed with the beeswax treated bran, confirmed by predictive analytical models and the DMTA results.

This work is innovative with respect to the literature on PHBV-based composites because it assesses whether natural waxes, in addition to increasing the biobased percentage of the formulation, are useful in improving adhesion and homogenizing the dispersion of waste fillers, such as bran, within a polyester matrix.

Indeed PHBV-based biocomposites could be used to manufacture molded items to give a new life to a waste residue and to yield biopolyester-based formulation at more affordable costs.

In conclusion, the addition to PHBV-based matrix of wheat bran powder, treated with beeswax, appears to be a method suitable to (i) valorize an abundant agro-food by-product such as wheat bran, (ii) favor the production of bio-composites with good mechanical properties valuable for practical applications, and (iii) reduce the cost of the final products based on PHBV.

## Figures and Tables

**Table 1:** Composition (wt %) of the PHBV/bran biocomposites.

Sample	PHBV	CaCO_3_	ATBC	Bran	T561 Wax	T581 Wax
Polymeric Matrix (M)	85	5	10	-	-	-
M + 5 bran (B5)	80.75	4.75	9.5	5	-	-
M + 10 bran (B10)	76.5	4.5	9	10	-	-
M + 15 bran (B15)	72.25	4.25	8.5	15	-	-
M + 5 bran T561 (B5_T561)	80.75	4.75	9.5	4.75	0.25	-
M + 10 bran T561 (B10_T561)	76.5	4.5	9	9.5	0.5	-
M + 15 bran T561 (B15_ T561)	72.25	4.25	8.5	14.25	0.75	-
M + 5 bran T581 (B5_T581)	80.75	4.75	9.5	4.75	-	0.25
M + 10 bran T581 (B10_T581)	76.5	4.5	9	9.5	-	0.5
M + 15 bran T581 (B15_T581)	72.25	4.25	8.5	14.25	-	0.75

**Table 2:** Enthalpy of melting (Δ*h_m_*), and crystalline weight fraction (*w_C_*) of the as prepared PHBV based matrix and biocomposites (estimated errors: ±1 J/g for Δ*h_m_*, and ±0.02 for *w_C_*)

Sample	Δ*h_m_* (J/g)	*w_C_*
M	94	0.66
B10	91	0.64
B10_T561	93	0.65
B10_T581	91	0.64

**Table 3:** *T*_g_ values, Storage modulus (E’) at 25 °C and adhesion factor (A) at 25 °C of composites with 10% bran

Sample	*T*_g_ (°C)	E’ (MPa)	A (-)
M	4	1912	0
B10	9	2190	−0.257
B10_T561	8	2101	−0.374
B10_T581	8	2020	−0.095
